# Systematic review of renal denervation for the management of cardiac arrhythmias

**DOI:** 10.1007/s00392-021-01950-8

**Published:** 2021-11-08

**Authors:** Nakulan Nantha Kumar, Kuda Nyatsuro, Shiraz Ahmad, Ibrahim T. Fazmin, Khalil Saadeh, Gary Tse, Kamalan Jeevaratnam

**Affiliations:** 1 Bristol Medical School, University of Bristol, Bristol, UK; 2 Faculty of Health and Medical Sciences, University of Surrey, Guildford, GU2 7AL UK; 3 Royal Papworth Hospital NHS Foundation Trust, Cambridge, UK; 4 School of Clinical Medicine, University of Cambridge, Cambridge, UK; 5 Tianjin Key Laboratory of Ionic-Molecular Function of Cardiovascular Disease, Department of Cardiology, Tianjin Institute of Cardiology, Second Hospital of Tianjin Medical University, Tianjin, 300211 China

**Keywords:** Renal sympathetic denervation (RSD), Cardiac arrhythmia, Atrial fibrillation (AF), Cardiac electrophysiology

## Abstract

**Background:**

In the wake of the controversy surrounding the SYMPLICITY HTN-3 trial and data from subsequent trials, this review aims to perform an updated and more comprehensive review of the impact of renal sympathetic denervation on cardiac arrhythmias.

**Methods and results:**

A systematic search was performed using the Medline, Scopus and Embase databases using the terms “Renal Denervation” AND “Arrhythmias or Atrial or Ventricular”, limited to Human and English language studies within the last 10 years. This search yielded 19 relevant studies (*n* = 6 randomised controlled trials, *n* = 13 non-randomised cohort studies) which comprised 783 patients. The studies show RSD is a safe procedure, not associated with increases in complications or mortality post-procedure. Importantly, there is no evidence RSD is associated with a deterioration in renal function, even in patients with chronic kidney disease. RSD with or without adjunctive pulmonary vein isolation (PVI) is associated with improvements in freedom from atrial fibrillation (AF), premature atrial complexes (PACs), ventricular arrhythmias and other echocardiographic parameters. Significant reductions in ambulatory and office blood pressure were also observed in the majority of studies.

**Conclusion:**

This review provides evidence based on original research that ‘second generation’ RSD is safe and is associated with reductions in short-term blood pressure and AF burden. However, the authors cannot draw firm conclusions with regards to less prominent arrhythmia subtypes due to the paucity of evidence available. Large multi-centre RCTs investigating the role of RSD are necessary to comprehensively assess the efficacy of the procedure treating various arrhythmias.

**Graphic abstract:**

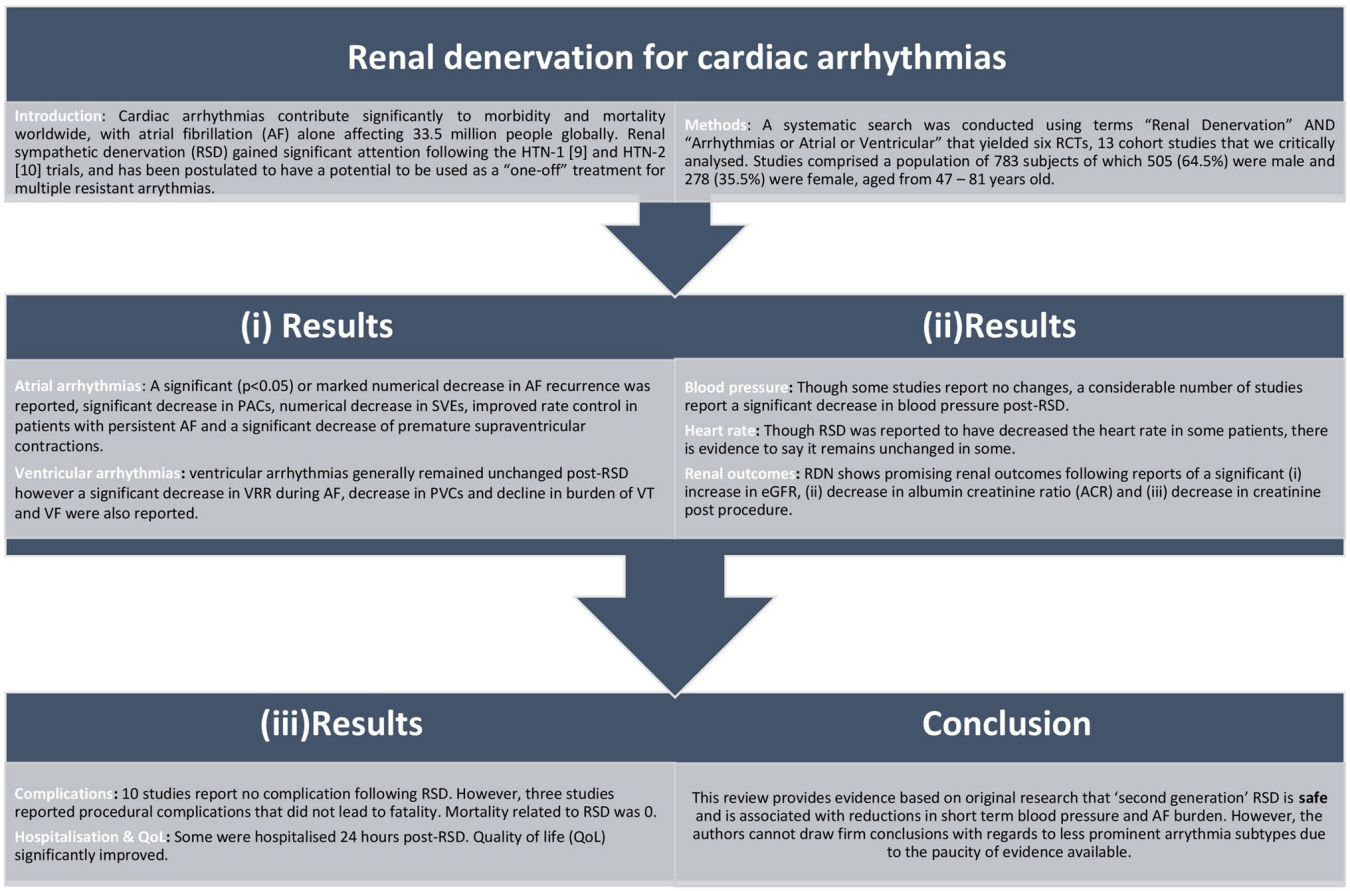

## Introduction

Cardiac arrhythmias contribute significantly to morbidity and mortality worldwide [1, 2]. Atrial arrhythmias are the commonest arrhythmias observed in clinical practice with an increasing prevalence due partly to an ageing population, with atrial fibrillation (AF) alone affecting 33.5 million people globally [1]. AF is additionally linked with strokes which can be highly debilitating [3, 4]. By contrast, ventricular arrhythmias are less common but are associated with sudden cardiac death [5], affecting around 184,000–450,000 individuals per year in the United States of America [2, 6, 7].

Arrhythmias are complex biophysical phenomena, and despite significant advances in medical technology over the past decades, current medical and invasive management options cannot abolish their occurrences completely. Renal sympathetic denervation (RSD) was first performed in 1925 [8], but due to its inability to reduce blood pressure safely as well as effectively in patients with resistant hypertension, did not gain significant attention until the recent consequential findings of the HTN-1 [9] and HTN-2 [10] trials. Given that hypertension is one of the most important risk factors for developing AF and its recurrence [11], there has been speculation that RSD may reduce AF burden [12, 13]. Whilst this may be a direct consequence of BP reduction, there is some evidence that the impact of RSD on cardiac arrhythmias may be independent of BP effects [14]. RSD has also proven to have a role in several related conditions in which the autonomic nervous system plays an important role, such as heart failure, chronic kidney disease (CKD) and glucose metabolism [8, 15]. Thus, RSD empirically has a plausible biological mechanism to affect the autonomic nervous system, feedback onto the cardiovascular system, and together with early studies [14] hinting at a possible antiarrhythmic effect. Thus, the results of HTN-3 trial [16] that found that both the RSD and control groups showed similar BP reductions at 6 months, do not directly affect the potential of RSD in treating cardiac arrhythmias. The data from the HTN-3 [16] trial have been reported to be controversial for the following reasons: (1) medication adherence (2) suboptimal procedure performance, (3) patient selection and (4) operator experience [17–19]. Since then, there have been many studies such as the DENERHTN trial [20], the SPYRAL HTN-OFF MED trial [21], RADIANCE-HTN SOLO trial [22] and SPYRAL HTN-ON MED [23] trial that report a significant reduction in BP.

In 2016, a systematic review examined the effects of RSD on cardiac arrhythmias [14]. However, since then, additional trials have been completed and published. Moreover, the review did not examine all domains relevant to RSD and cardiac arrhythmias. For example, whilst there is a relatively larger body of evidence on the short- and long-term effects of RSD on patients with AF and hypertension, the effects of RSD on AF in normotensive patients or on ventricular arrhythmias have yet to be fully investigated. In this study, we aim to perform an updated and comprehensive systematic review on the effects of RSD on both atrial and ventricular arrhythmias.

## Methods

### Search strategy

**Fig. 1:**
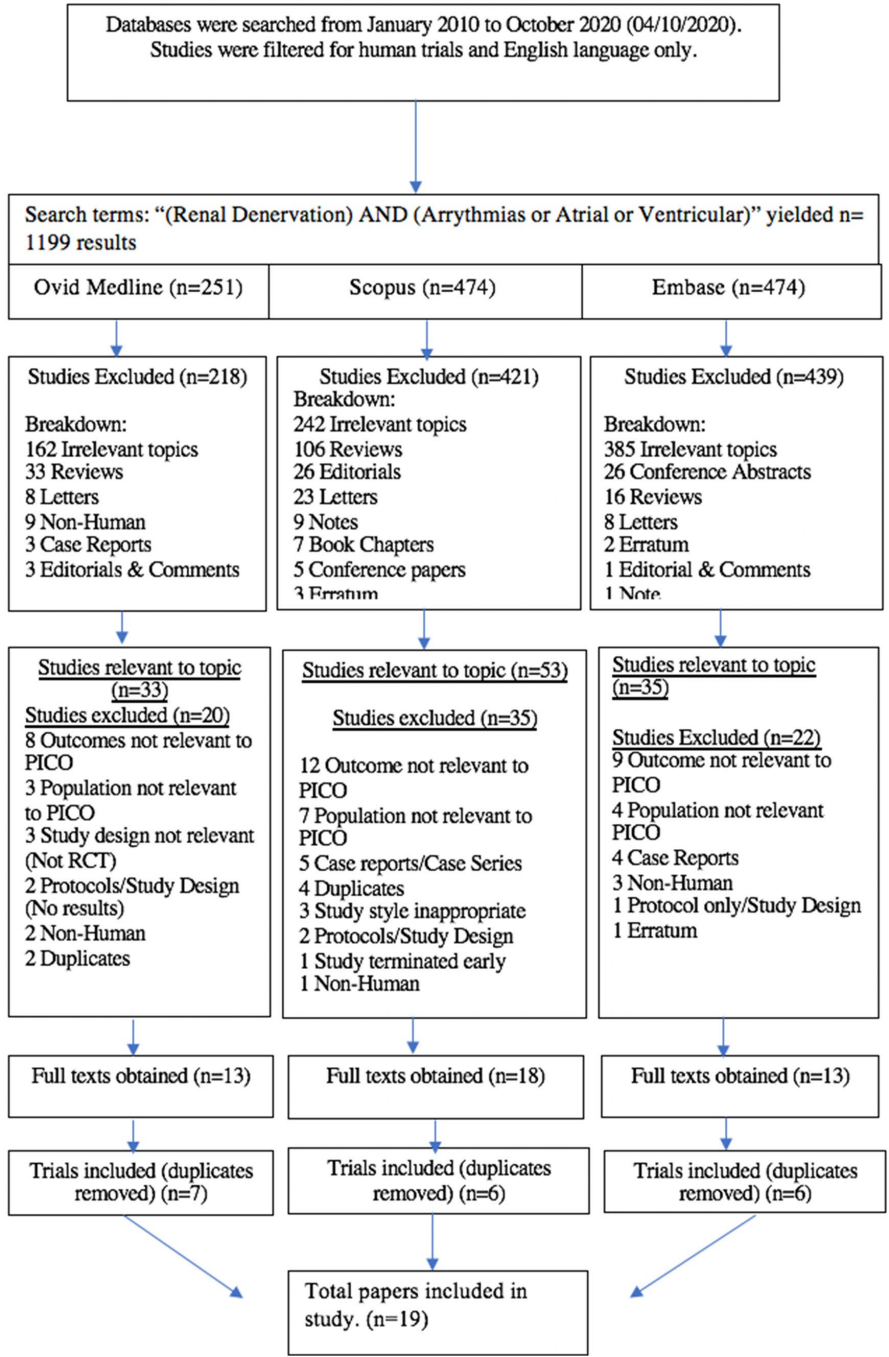
Search strategy. *PICO* population, intervention, comparison and outcomes

This review used Preferred Reporting Items for Systematic Reviews and Meta-Analyses (PRISMA). Medline, Scopus and Embase were searched from January 2010 to October 2020 using search terms “Renal Denervation” AND “Arrhythmias or Atrial or Ventricular”. Searches were limited to that of original research and human studies in the English language. One reviewer (KN) independently screened the titles and abstracts of the results obtained from the search strategy. From the initial 1192 papers initially identified by the search strategy (Fig. 1), 44 relevant studies were selected and full texts obtained. Three independent reviewers (NNK, KN and KJ) then independently studied full texts to include and exclude relevant studies based on the exclusion criteria defined in the protocol. The reference lists of these studies were studied to identify other relevant studies to be included in the studies. Duplicates were also screened for and removed as appropriate. Any discrepancies were discussed and resolved internally with the senior author (KJ).

### Extraction

Extraction was then conducted by one of the reviewers (NNK) onto a standardised excel extraction sheet independently. The reviewer extracted all outcomes listed in the inclusion criteria. Two other reviewers (KN and KJ) independently checked the extracted data with the original full texts.

### Inclusion criteria

We included studies that were (1) original research study, i.e. randomised controlled trial (RCT) or non-randomised observational studies (retrospective or prospective cohort), (2) studied a human population with any form of cardiac arrhythmia, (3) had a treatment arm where RSD was used, (4) reported outcomes as follows: arrhythmia (atrial or ventricular) recurrence, changes in blood pressure, change in heart rate, mortality, hospitalisation, complications (general or procedural), renal function and cardiac morphological changes (echocardiographic parameters).

## Results

### Search results and study characteristics

**Fig. 2:**
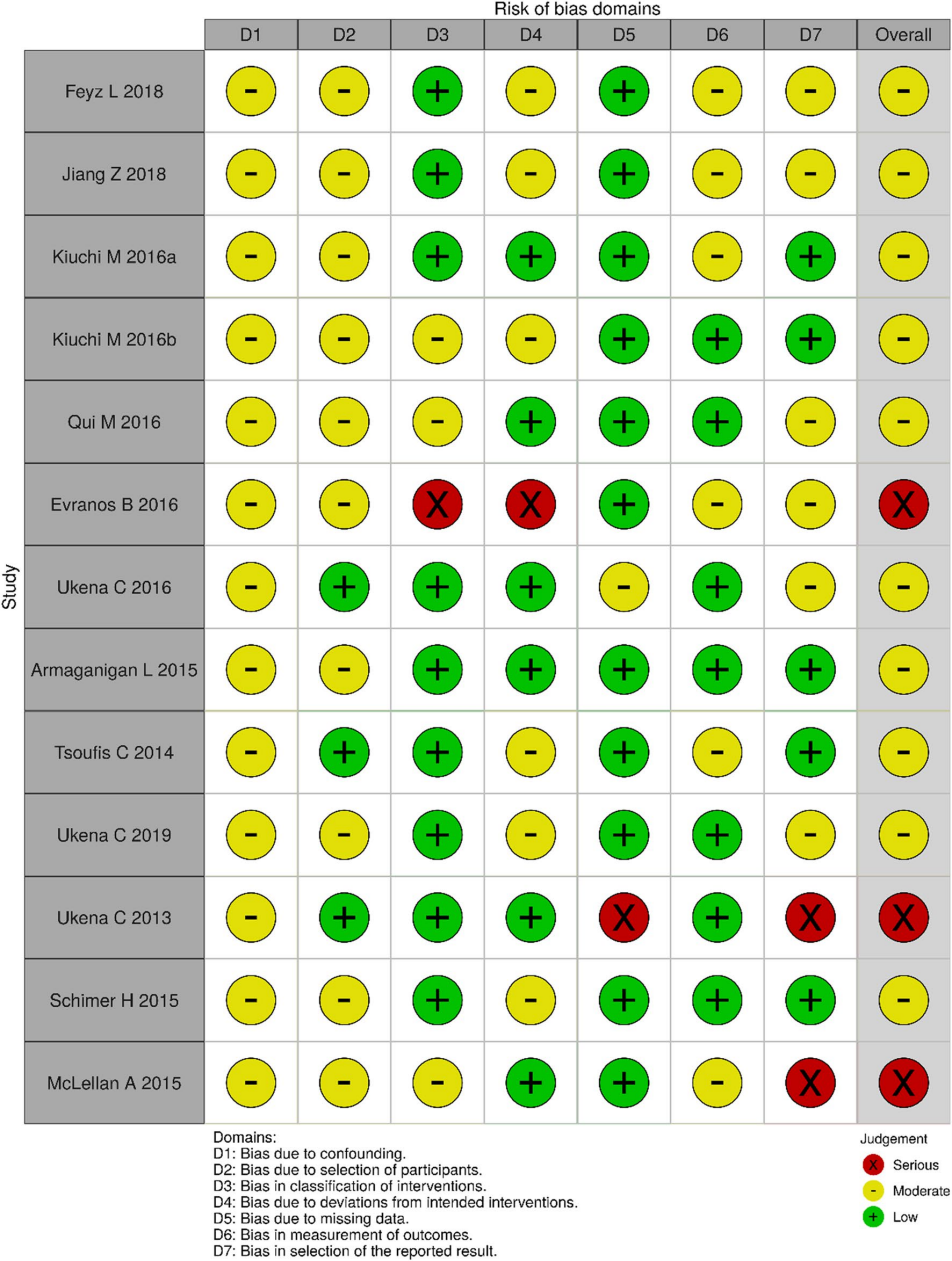
ROBINS-I evaluation of included studies. *ROBINS-I* risk of bias in non-randomised studies-of interventions

**Fig. 3:**
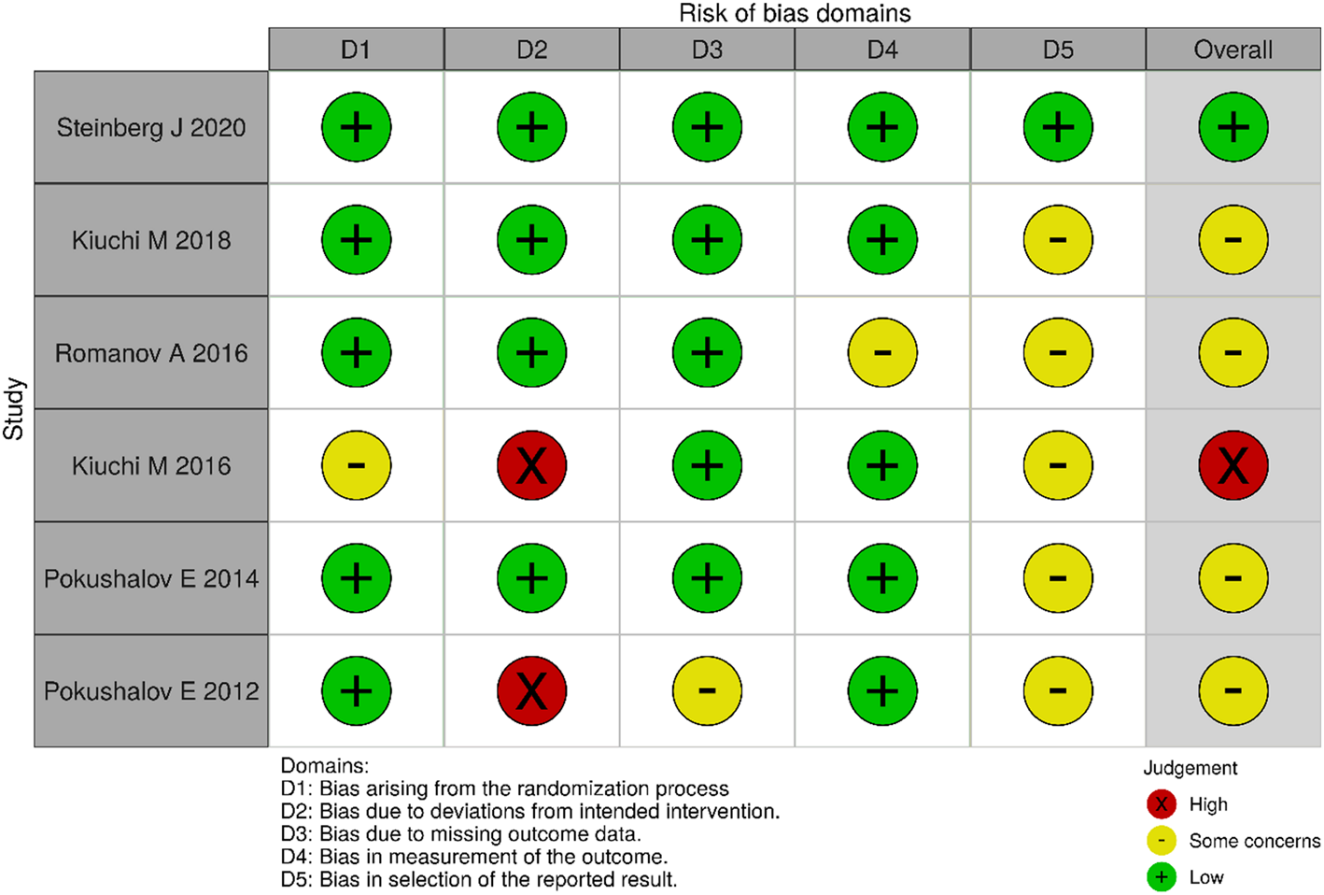
RoB-2 evaluation of included studies. *RoB-2* risk of bias-2

The search was conducted on the fourth of October 2020 and yielded a total of 1199 results across the Ovid Medline, Scopus and Embase databases. 1078 studies were excluded after screening of titles and abstracts due to irrelevant study design or topic. Of the remaining *n* = 121 studies, *n* = 77 were excluded (*n* = 29 due to irrelevant outcomes, *n* = 14 irrelevant population, *n* = 11 irrelevant study design or had no results published at the time of search, *n* = 9 case reports, *n* = 6 duplicates, *n* = 1 study was terminated early, and *n* = 1 erratum). Forty-four full-text studies were obtained and assessed by 2 reviewers (NNK, KN). A further 25 studies were excluded as duplicates, resulting in a total of 19 studies included in this review (Fig. 1). These 19 studies comprised 6 randomised controlled trials (RCTs) and 13 were non-randomised cohort studies. The population across all 19 studies was 783 participants of which 505 (64.5%) were male and 278 (35.5%) were female. The age across the studies included ranged from 47 to 81 years (Table 1). Risk of bias was assessed using the Risk of Bias in Non-randomised Studies—of Interventions (ROBINS-I) tool for non-randomised studies (Fig. 2) and the Revised Cochrane risk of bias tool for randomised trails (RoB-2) for randomised trials (Fig. 3).

**Table 1:** Study demographics

Author	Year	Age	Body mass index (kg/m^2^)	Male	Female	Country	Design	Population sample size (RSD cases, n)	Intervention	Cardiac disease	Comparator arm
Steinberg [30]	2020	Median 59 (IQR) (54–65)	NR	91	63	Russian Federation, Poland, and Germany	RCT	154	PVI + RSD	Paroxysmal AF and HTN	PVI only
Ukena [33]	2019	63.5 ± 10	NR	70	35	Germany	Prospective cohort study	105	RSD	Resistant HTN, premature atrial (PAC) and ventricular captions (PVC)	N/A
Feyz [31]	2018	64 ± 7	30.7 ± 5.6	9	11	Netherlands	Prospective cohort study	20	RSD	Paroxysmal and persistent AF, primary HTN	N/A
Kiuchi [26]	2018	56.8 ± 6.5	27.1 ± 1.9	25 (76%)	8	Brazil	RCT	33	PVI + RSD in CKD patients	Paroxysmal and drug refractory AF, HTN in chronic kidney disease (CKD) patients	PVI + spironolactone
Jiang [37]	2018	51.4 ± 14.3	NR	7	1	China	Prospective cohort study	8	RSD	Electrical storm and recurrent ventricular arrhythmia	N/A
Romanov [32]	2016 (made available 2017)	56 ± 6	NR	29	10	Russia, New York, NY, USA	RCT	39	PVI + RSD	Persistent and paroxysmal AF, drug-resistant HTN	PVI only
Kiuchi [25]	2016	60 ± 14	24.9 ± 4.4	24	15	Brazil	Prospective cohort study	39	PVI + RSD	Controlled HTN and paroxysmal AF in CKD patients	PVI only
Kiuchi [24]	2016	52.3 ± 11.4	26.3 ± 2.6	15	5	Brazil	Prospective cohort study	20	RSD	Basic or polymorphic premature ventricular complexes (PVCs)	Sham trial (Control)
Qiu [34]	2016	57.5 ± 10.2 (subgroups 51.9 ± 10.7; 58.4 ± 10.1; 62.1 ± 8.4)	27.1 ± 1.4, 24.9 ± 2.9, 25.3 ± 2.7	16	5	China	Prospective cohort study	21	RSD	Symptomatic, persistent AF and HTN	N/A
Evranos [38]	2016	47–81 (range)	NR	12	4	Turkey	Retrospective cohort study	16 (see results, table last page)	RSD in adjunct to catheter ablation	Ventricular Arrhythmia (VA), in patients with dilated cardiomyopathy	N/A
Ukena [39]	2016	59.2 ± 14.4	30.2 ± 7.7	13	0	5 centres in Australia and Europe	Retrospective cohort study	13	PVI + RSD (catheter-based)	Ventricular arrhythmias in heart failure (HF) patients	N/A
Kiuchi [27]	2016	68 ± 9	27 ± 3	13	8	Brazil	RCT	21	PVI + RSD	Paroxysmal and persistent AF, HTN, in CKD patients	PVI only
Schirmer [41]	2015	63.5 ± 1.2	29.4 ± 0.6	36	30	Germany	Prospective cohort study	66	RSD	PAC, resistant HTN	N/A
Armaganijan [40]	2015	64.5 ± 6.3	NR	5	5	Brazil	Prospective cohort study	10	RSD	Refractory ventricular arrhythmia (VT and VF)	N/A
McLellan [35]	2015	64 ± 9	31 ± 3	10	4	Australia	Prospective cohort study	14	RSD	HTN; atrial and ventricular arrhythmias	N/A
Tsioufis [36]	2014	55.4 ± 8.9	33.7 ± 5.7	9	5	USA	Prospective cohort study	14	RSD	Drug-resistant HTN; atrial and ventricular arrhythmias	N/A
Pokushalo[29]	2014	56 ± 6	NR	31	10	USA	RCT	41	PVI + RSD	AF and HTN	PVI only
Ukena [42]	2013	62.2 ± 0.8 years	31.4 ± 0.4	79	57	Germany	Prospective cohort study	136	RSD	Resistant HTN, atrioventricular (AV) conduction	N/A
Pokushalov [28]	2012	57 ± 8	28 ± 6	11	2	Russia	RCT	13	PVI + RSD	AF (paroxysmal or persistent AF), drug-resistant HTN	PVI only

Study demographics. Age has been reported as mean ± SD or stated otherwise

*AF* atrial fibrillation, *CKD* chronic kidney disease, *HTN* hypertension, *NR* not reported, *PAC* premature atrial complex, *PVC* premature ventricular complex, *PVI* pulmonary vein isolation, *RCT* randomised clinical trial, *RSD* renal sympathetic denervation, *VF* ventricular fibrillation

### Recurrence of atrial arrhythmias

Fourteen studies included atrial arrhythmia outcomes such as AF, atrial flutter, premature atrial contractions (PACs), supraventricular ectopic beats (SVEs), atrial premature complexes and premature supraventricular contractions. Thirteen studies studied concurrent RSD (with or without PVI), and 1 [24] of the studies being crossover/RSD only*.* At 12 months post-RSD, eight studies report recurrence of AF as “freedom from AF”, “AF-free”, “total AF episodes” and “AF burden (min/day).” Of the 18 studies that investigated the recurrence of arrhythmias (atria and ventricle) post-RSD, the majority of used 24-Holter monitors (*n* = 9) to capture the episode of recurrence at defined time points. Four studies (*n* = 4) used implantable cardiac defibrillators (ICDs), one study (*n* = 1) used an implantable cardiac monitor, one study (*n* = 1) used ECG, one study (*n* = 1) used a pacemaker, one study (*n* = 1) used “electrophysiology studies” and one study (*n* = 1) used a 7-day Holter monitor.

However, it is worth nothing that one of these studies reports AF recurrence which is not exclusively 12 months and 22.4 ± 12.1 months was the follow-up period [25]. In these eight studies, six of them compared pulmonary vein isolation in the treatment of AF with pulmonary vein isolation and RSD. In the remaining two studies, the only invasive intervention was the RSD itself (Table 1). All studies used Holter monitoring to detect the presence of AF at the pre-defined follow-up points (from 1 to 12 months) though the total time under surveillance was different. Four of these studies used Holter monitoring varying from 24 h to 7 days at the pre-defined intervals to detect AF. The remaining studies used implantable cardiac monitors to continuously monitor for arrhythmias or used implantable pacemakers to detect arrhythmias. Four studies reported “freedom from AF” at 12 months as 61% (*n* = 20), 76% (*n* = 16), 69% (*n* = 9) and 63% (*n* = 26), respectively [26–29]. All studies, whether RSD alone or RSD with PVI, report a significant (p < 0.05) or marked numerical decrease in AF recurrence, or a higher number of patients that are AF free in the treatment arm that includes RSD as opposed to the control group [25–32]. In addition to freedom from AF, Steinberg et al. [1] reported freedom from atrial flutter and tachycardia at 12 months that was significantly higher in the RSD treatment group (p = 0.006).

The remaining studies looked into outcomes of various other types of rhythm abnormalities with significant findings. Ukena et al. [33] and Schirmer et al. reported a significant decrease in PACs at 6 months. Feyz et al. [31] reported a numerical decrease in SVEs at 6 and 12 months post-RSD. Some studies looked at more short-term outcomes for various atrial arrhythmias. Qiu et al. [34] reported that RSD was postulated to have improved rate control in patients with persistent AF at 7 days post-procedure. McLellan et al. [35] reported there not to be any change at 7 days post-RSD with regards to PACs and atrial tachyarrhythmias. Tsioufis et al. [36] reported a significant decrease of premature supraventricular contractions at 1 and 6 months post-RSD.

### Recurrence of ventricular arrhythmias

Nine studies investigated the impact of RSD on different ventricular arrhythmia outcomes, such as premature ventricular contractions (PVCs), ventricular ectopic beats (VE), ventricular rate response (VRR), “median ventricular arrhythmia episodes per month” that included episodes of electrical storm (ES) [37], polymorphic premature ventricular complexes (PVCs), burden or episodes of ventricular tachycardia (VT) and ventricular fibrillation (VF). Three studies report that various subtypes of ventricular arrhythmias remained unchanged post-RSD at different time points (7 days, 6 months and 12 months) [31, 33, 35]. However, Feyz et al. [31] reported a significant decrease in VRR during AF at 6 and 12 months, and Jiang et al. [37] reported a significant decrease in media ventricular arrhythmia episodes per month. Kiuchi et al. [24] reported a significant decrease in PVCs at 1 and 6 months after RSD. Evranos et al. [38] report a significant decline in burden of VT and VF at 3, 6, 12 and 18 months. Ukena et al. [39] in an earlier study also report a significant decrease in VT and VF at 1 and 3 months. Armaganijan et al. [40] report a reduced median number of VT and VF at 1 and 6 months. Tsoufis et al. [36] report a significant decrease in PVC and “ventricular arrhythmias” at 1- and 6-month follow-ups (Table 2).

**Table 2:** Arrhythmia outcomes

Author	Year	Atrial arrhythmia outcomes	Ventricular arrhythmia outcomes
Steinberg [30]	2020	The study reported freedom from AF, atrial flutter, or atrial tachycardia at 12 months in 111 of 154 patients who underwent RSD + PVI (72.1%) with a p value of 0.006 compared to the control group, which used PVI only (hazard ratio, 0.57; 95% CI 0.38–0.85; *p* = 0.006)	NR
Ukena [33]	2019	The study found that in a subgroup of patients, *n* = 20 who had ≥ 6 premature atrial contraction (PAC) per hour at baseline, there was a significant decrease in PAC burden at 6 months [median change − 12.4 (− 37.4 to − 2.3)], *p* = 0.002. On the whole in *n* = 77, with a baseline PAC/h 1.2 (0.3–6.2), there was no change in PACs, in 6 months, with a reading of 1.2 (0.2–4.2), *p* = 0.201	Premature ventricular contractions (PVC)/h were reported to be 1.2 (0.1–13.9) at baseline in *n* = 77 and were 0.9 (0.1–10.5) at 6 months. It was reported to have remained unchanged at 6 months, *p* = 0.619
Feyz [31]	2018	Total AF episodes (min) decreased from 125 (2–978) at pre-RSD to 44 (0–2833) at 6 months (*p* = 0.64) and 84 (0–544) at 12 months (*p* = 0.03). It was reported that AF burden (min/day) decreased from pre-RSD at a median (IQR) of 1.39 (0–11) to 0.67 (0–31.6) at 6 months with *p* = 0.64. AF burden further decreased to 0.94 (0–6.0) at 12 months (*p* = 0.03, when pre-RSD is compared with 12 months). There was a numerical decrease in supraventricular ectopic beats (SVEs) at 6- and 12-month follow-up, with p values of 0.57 and 0.73, respectively	Ventricular ectopic beats remained unchanged from a baseline of 35 (3–153) to 22 (3–86) at 6 months and 42 (5–134) at 12 months with p values 0.57 and 0.73, respectively. The highest ventricular rate response (VRR) during AF was said to decrease at 6 and 12 months with a p value of *p* = 0.09 and *p* = 0.01, respectively
Kiuchi [26]	2018	There was a significantly higher number (*n* = 20) of patients who were AF free at 12 months in the RSD + PVI group (61%), in comparison to the comparison group, *p* = 0.0242. Mean AF burden was significantly lower in the PVI + RSD group as compared to comparison group, after 9 months: Δ = − 10%, and after 12 months: Δ = − 12%, with *p* < 0.0001 for both. However, it is reported that no changes were noted in the mean AF burden from the 3rd month to 6, 9, and 12 months in the PVI + RSD group	NR
Jiang [37]	2018	NR	Median ventricular arrhythmia (VA) episodes per month were reported to have significantly decreased from 3.17 (range 0.33–15.33) to 0.10 (range 0–5.83), *p* < 0.05. In the first 3 months post-RSD, *n* = 2 patients had an episode of electrical storm with 30 episodes and 60 episodes of ventricular tachycardia/fibrillation. 3 months after RSD, all patients were free from electrical storm
Romanov [32]	2016	RSD + PVI was reported to have significantly decreased AF recurrences burden. Freedom from AF recurrence was 0.61 (95% confidence interval, CI: 0.51–0.81) in the RSD group at 12 months	NR
Kiuchi [25]	2016	AF recurrence was lower in the RSD + PVI group compared to the control (PVI only) group in the 22.4 ± 12.1-month follow-up of CKD patients, *p* = 0.0251. RSD + PVI significantly reduced AF recurrence in CKD stage 4 patients	NR
Kiuchi [24]	2016		Polymorphic premature ventricular complexes (PVCs) in the RSD group were reported to have significantly decreased from baseline to 3, 6, 7 and 12 months, with readings of 36,091 ± 3327, 31,009 ± 3251, 20,411 ± 3820, 7701 ± 1549, and 1274 ± 749, respectively. *p* < 0.0001 for all. There was a significant difference in the decline of PVCs in the RSD vs control group at 7 and 12 months, *p* < 0.001
Qiu [34]	2016	RSD improved rate control in patients with persistent AF at 7 days, *p* > 0.001	NR
Evranos [38]	2016	NR	The study reported a significant decline in the burden of ventricular tachycardia (VT) and fibrillation (VF), anti-tachycardia pacing (ATP) and shock therapies delivered from ICDs in the RSD group, *p* < 0.05, during the follow-up (3, 6, 12 and 18 months)
Ukena [39]	2016	NR	Episodes of ventricular tachycardia (VT) and fibrillation (VF) decreased to 2 (0–7) and 0, at 1 and 3 months post-RSD with p values of 0.004 and 0.006, respectively. The number of ICD intervention also declined. ATP reduced from 2 (0–7) (*p* = 0.004) and 0 (*p* = 0.006), and ICD shocks from 14 (9–30) to 1 (0–7) at 1 month (*p* = 0.004). 1 month post-RSD, *n* = 11 (85%) patients were completely free from VT and VF
Kiuchi [27]	2016	The RSD + PVI group had significantly more patients (*n* = 16, 76%) that were free from AF at 12 months compared to the control group, *p* = 0.0007. At 12 months, only *n* = 5 (24%) of patients in RSD group had AF recurrence	NR
Schirmer [41]	2015	Premature atrial contractions (PAC) of median of > 153 PAC/24 h was reduced at 6 months to 68 PAC/24 h post-RSD, *p* = 0.032	NR
Armaganijan [40]	2015	NR	Median number of VT/VF episodes, anti-tachycardia pacing, shocks was reduced from baseline to 1 (range 0–17)/0 (range 0–7)/0 (range 0–3) at 1 month and 0 (range 0–9)/0 (range 0–7)/0 (range 0–3) at 6 months post-RSD. Two patients sustained VT with the first week but no further cases after that during the follow-up
McLellan [35]	2015	There was no change in burden of atrial premature complexes/24 h (*p* = 0.79), no sustained (or sustained) atrial tachyarrhythmias at 7 days post-RSD	There was no change in burden of ventricular premature/24 h(*p* = 0.22) complexes, non-sustained (or sustained) ventricular tachyarrhythmias(*p* = 1.0) at 7 days post-RSD
Tsioufis [36]	2014	Premature supraventricular contractions significantly decreased 1 month (*p* = 0.039) and 6 months (*p* = 0.044) post-RSD	Ventricular contractions significantly decreased. Complex ventricular arrhythmias were present in *n* = 5 at baseline and reduced to *n* = 2 at 1 month post-RSD, and *n* = 1 at 6 months. The number of premature ventricular contractions (PVC) significantly decreased at 1 month and 6 months (*p* = 0.001) post-RSD
Pokushalov [29]	2014	There was a significantly higher number, *n* = 26 (63%) of patients that were AF free in the RSD + PVI group compared to the control group, at 12 months, *p* = 0.014	NR
Ukena [42]	2013	The study reported an increase in PR interval at 3 and 6 months that was significant. 57% of the patients (*n* = 72) had a PR prolongation of ≥ 10 ms. Initially, 15% of patients met the criteria of 1st-degree atrioventricular block (AVB) but this increased significantly to 32% at 3 months and 26% at 6 months(*p* > 0.001). ECG parameters such as QRS or QTc were reported to have not been affected	NR
Pokushalov [28]	2012	It is reported that *n* = 9 patients (69%) in the RSD + PVI group were AF free at 12 months in contrast to only *n* = 4 (29%) in the control group, *p* = 0.033	NR

*AF* atrial fibrillation, *ATP* anti-tachycardia pacing, *CKD* chronic kidney disease, *HTN* hypertension, *NR* not reported, *PAC* premature atrial complex, *PVC* premature ventricular complex, *PVI* pulmonary vein isolation, *RCT* randomised clinical trial, *RSD* renal sympathetic denervation, *VF* ventricular fibrillation

### Changes in heart rate

Eleven studies reported the effects of RSD on HR at various time points after RSD. Six trials reported HR to have remained unchanged or have a non-significant change at 7 days and 1, 6 and 12 months post-RSD [25, 31, 33, 37, 39, 40]. However, in a trial by Ukena et al. [33], there is a subgroup of patients with a baseline HR of > 72 bpm, that have been reported to have had a significant reduction in HR at 6 months. Similarly, there are five other studies that have documented a significant decrease in HR post-RSD at various time points during follow-up (7 days, 1, 3 and 6 months) [24, 34, 36, 41, 42]. Furthermore, Kiuchi et al. [24] reported a numerical decrease in HR at 1 and 6 months post-RSD (Table 3).

**Table 3:** Heart rate and blood pressure outcomes

Author	Year	Heart rate (HR) outcomes (bpm)	Blood pressure (BP) outcomes (mmHg)
Steinberg [30]	2020	NR	Patients in the PVI + RSD group experienced a significant decrease in both mean SBP and mean DBP at 12 months. The decrease in SBP was from 150 mmHg (95% CI 149–152 mmHg) to 135 mmHg (95% CI 133–136 mmHg), for a mean reduction of 16 mmHg (95% CI 14–18 mmHg; *p* < 0.001), and the decrease in DBP was from 150 mmHg (95% CI 149–152 mmHg) to 135 mmHg (95% CI 133–136 mmHg), for a mean reduction of 16 mmHg (95% CI 14–18 mmHg; *p* < 0.001)
Ukena [33]	2019	Average HR did not change significantly. Baseline mean 24-h HR was 65.7 ± 9.9 bpm and 65.3 ± 10 at 6 months (*p* = 0.772). However, there were a subgroup of patients who had a baseline 24-h HR of > 72 bpm that exhibited a significant reduction by 2.31 ± 7.1 bpm at 6 months (*p* = 0.042)	24-h and Office BP both significantly reduced at 6 months from a mean BP of 171.1 ± 24.6/91.5 ± 15 mmHg at baseline. 24-h SBP was reduced by 7.8 ± 18.6 (*p* < 0.001) and 24-h DBP by 3.7 ± 11.1 (*p* = 0.001) mmHg. Office SBP decreased by 21.8 ± 25.2 mmHg and office DBP by 8 ± 18.7 mmHg (*p* < 0.001)
Feyz [31]	2018	Mean 24-h HR remained unchanged through follow-up with a mean of 66 ± 8 bpm at 6 months (− 5 ± 14 bpm; *p* = 0.15) and mean of 70 ± 12 bpm at 12 months (− 1 ± 14 bpm; *p* = 0.63) from a baseline mean of 71 ± 15 bpm	Office SBP was reported to have significantly decreased at 12 months (*p* < 0.01) to 133 ± 16 mmHg from a baseline of 153 ± 17 mmHg. Other measurements of BP showed a numerical decrease in BP. Office SBP at 6 months was reported to be 148 ± 17 mmHg (*p* = 0.13). Office DBP reduced from 89 ± 10 mmHg to 81 ± 1 mmHg (*p* = 0.006) and 81 ± 10(*p* = 0.007) mmHg at 6 and 12 months, respectively. 24-h BP (SBP/DBP) decreased from a baseline of 131 ± 16/78 ± 9 mmHg to 121 ± 9 (*p* = 0.007)/72 ± 6 (*p* = 0.006) and 124 ± 11 (*p* = 0.07)/74 ± 9 (*p* = 0.16) mmHg at 6 and 12 months, respectively
Kiuchi [26]	2018	NR	The baseline 24-h SBP of 142 ± 6 mmHg decreased significantly to 132 ± 5 (*p* < 0.0001) and 123 ± 4 (*p* < 0.0001) mmHg at 6 and 12 months, respectively. The 24-h DBP decreased significantly from a baseline of 103 ± 8 mmHg to 95 ± 8 (*p* < 0.0001) at 6 months and 82 ± 4 (*p* < 0.0001) mmHg at 12 months
Jiang [37]	2018	Pre-RSD HR of 69.3 ± 13.9 unchanged at 6 months and recorded as 70.6 ± 14 bpm (*p* = 0.395)	Baseline BP (SBP/DBP) of 111.5 ± 8.9/70.6 ± 7.7 mmHg remained unchanged at 6 months and was recorded as 103.8 ± 12.4/67.9 ± 7.1, *p* = 0.262/*p* = 0.482
Romanov [32]	2016	NR	A baseline BP (SBP/DBP) of 163 ± 20/88 ± 13 mmHg was reduced to a mean BP 104 (95% CI: 103–106) mmHg at 12 months. A substantially lower mean BP was achieved in the RSD group as opposed to the control group (PV1-only) at 12 months (no p value). 46% (*n* = 18) of the RSD patients achieved a target BP of < 140/90 mmHg as opposed to 0% in the control group
Kiuchi [25]	2016	Baseline average 24-h HR of 76 ± 16 was recorded as 73 ± 18 at 6 months. This was not a significant change in HR *p* = 0.0785	Mean systolic 24-h SBP/DBP at baseline was recorded as 121 ± 9/79 ± 6 and was recorded at 118 ± 7/78 ± 3 at 6 months. It is reported that there is no significant change in BP with p values *p* = 0.1673/0.4110
Kiuchi [24]	2016	Baseline mean 24-h HR of 78.7 ± 3.8 was reported as 70.3 ± 3.6 bpm (*p* < 0.0001) at 3 months, 46.4 ± 3.2 bpm (*p* < 0.0001) at 6 months, 75.8 ± 3.4 bpm (*p* = 0.1756) at 7 months and 76.3 ± 2.1 bpm (*p* = 0.2894) at 12 months. The decrease in HR was significant at 3 and 6 months. The change at 7 and 12 months were not significant	After adjustment of antiarrhythmic dosage or RSD, there was no significant change on 24-h BP in the 12 months of follow-up from baseline. Baseline of 122.7 ± 5.9/79.6 ± 3.2 was recorded and 122.3 ± 6.3/79.6 ± 4.1 at 3 months, 121.5 ± 5.3/79.1 ± 3.6 at 6 months, 121.2 ± 4.6/78.7 ± 4.3 at 7 months and 120.2 ± 4.0/78.8 ± 3.9 at 12 months
Qiu [34]	2016	Compared to baseline average HR, all 3 groups have a reduction of 22.6 ± 13.2 bpm (83.3 ± 4.9 vs 106.0 ± 14.6, *p* = 0.004), 9.7 ± 7.8 bpm (75.7 ± 7.6 vs 85.4 ± 3.7, *p* = 0.017) and 2.3 ± 2.9 bpm (71.4 ± 4.0 vs 73.7 ± 4.7, *p* = 0.089) at 7 days post-RSD. Maximum HR in 21 patients on Holter were all significantly reduced (76.8 ± 7.4 versus 88.4 ± 16.2, *p* < 0.001; 152.6 ± 24.7 versus 173.9 ± 37.7, *p* = 0.007) at 7 days post-RSD	No significant change was noted in 3 groups in office and 24-h BP when comparing baseline (Office SBP 125.1 ± 13.9, 118.0 ± 15.0, 127.8 ± 13.3; 24-h SBP 125.5 ± 18.7, 112.4 ± 17.9, 102.2 ± 12.81) (Office DBP 81.9 ± 9.8, 77.4 ± 9.1, 80.4 ± 7.8; 24-h DBP 81.3 ± 10.2, 77.2 ± 14.3, 65.6 ± 5.7) vs 7 days post-RSD (Office SBP 122.3 ± 9.9, 112.4 ± 12.2, 117.0 ± 14.5, Office DBP 79.1 ± 6.2, 67.6 ± 7.9, 77.8 ± 9.80 24-h SBP 115.5 ± 15.8, 110.6 ± 15.3, 100.6 ± 103 24-h DBP 77.3 ± 11.2, 75.0 ± 10.3, 62.4 ± 7.90) all p values > 0.01
Evranos [38]	2016	NR	Mean SBP was reported to be 120 ± 20 at baseline and 115 ± 15 at 15 months. This change was reported to be not significant, *p* > 0.05
Ukena [39]	2016	HR at baseline was 66.5 ± 13 and the change recorded at 1 month was not significant, *p* > 0.05	Blood pressure at baseline (SBP/DBP) was 115.9 ± 18/73.2 ± 12.9 and the change in both SBP and DBP at 1 month was reported to be not significant (*p* > 0.05 for both)
Kiuchi [27]	2016	NR	24-h BP (SBP/DBP) was reported as 119 ± 8/80 ± 3 at baseline, 115 ± 7/79 ± 3 at 3 months, 114 ± 7/78 ± 3 at 6 months and 114 ± 7/77 ± 3 at 12 months. There was no significant change in BP at all time points
Schirmer [41]	2015	HR of 67.7 ± 1.3 decreased significantly to 60.5 ± 1.2, an average decrease of 8.0 ± 1.3, *p* < 0.001 at 6 months	BP (SBP/DBP) significantly decreased from 172.9 ± 3.0/92.5 ± 2.3 mmHg to 151.3 ± 3.2/85.5 ± 1.6 mmHg (*p* < 0.001 for both) at 6 months
Armaganijan [40]	2015	NR	No significant changes were observed in SBP at 6 months, (mean SBP) 109.42 ± 19.32 from 113.57 ± 21.74 at baseline
McLellan [35]	2015	There was no significant change in 24-h HR from baseline 68 ± 11–7 days post-RSD 69 ± 8, *p* = 0.62	Mean 24-h BP (SBP/DBP) significantly reduced from 152/84 at baseline to 141/80 at 6 months, *p* < 0.01. Maximum 24-h BP reduced from 192/112 to 178/107 mmHg at 6 months, *p* = 0.03
Tsioufis [36]	2014	Mean 24-h HR decreased significantly by 6.7 bpm, *p* = 0.005 at 1 month and by 5.3 bpm, *p* = 0.006 at 6 months after RDN. Office HR portrayed a numerical decrease in 1 month by 6.9 bpm, *p* = 0.096 and a significant decrease by 10 bpm, *p* = 0.005 at 6 months	Office BP was reduced by 38/14 and 44/17, at 1 and 6 months, respectively, *p* = 0.001 for both. 24-h BP was reduced by 18/8.3 (*p* = 0.001/0.004) and by 20/10.3 (*p* = 0.002/0.005) at 1 and 6 months. Both Office BP and 24-h BP were reported to have decreased significantly at both 1 and 6 months
Pokushalov [29]	2014	NR	A significant decrease SBP and DBP was reported at time points 3, 6, 9 and 12 months. At 12 months, there was a significant decrease in BP –12.5 ± 7.8/7.8 ± 2.9 mmHg (*p* < 0.001 vs baseline) and –29.1 ± 14.6/–12.2 ± 7.7 mmHg (*p* < 0.001 vs baseline) in patients with moderate resistant HTN and severe resistant HTN, respectively
Ukena [42]	2013	A mean HR at baseline of 66.1 ± 1 was reduced by 2.6 ± 1 bpm (*p* = 0.001) and 2.1 ± 1.1 bpm (*p* = 0.046), at 3 and 6 months, respectively	Blood pressure was reduced significantly at 3 and 6 months post-RSD, from baseline (SBP/DBP) 176.7 ± 1.8/93.2 ± 1.3. SBP was reduced by 25.5 ± 2.4 and 28.1 ± 3 after 3 and 6 months (*p* ≤ 0.0001 for both) and DBP by 8.5 ± 1.5 and 10.5 ± 1.6 (both *p* ≤ 0.0001), respectively
Pokushalov [28]	2012	NR	A significant decrease in SBP/DBP was reported at time points 3, 6, 9, and 12 months. At 12 months, the SBP and DBP was significantly decreased compared to the control group (PVI only)

*AF* atrial fibrillation, *ATP* anti-tachycardia pacing, *CKD* chronic kidney disease, *DBP* diastolic blood pressure, *HTN* hypertension, *NR* not reported, *PAC* premature atrial complex, *PVC* premature ventricular complex, *PVI* pulmonary vein isolation, *SBP* systolic blood pressure, *RCT* randomised clinical trial, *RSD* renal sympathetic denervation, *VF* ventricular fibrillation

### Blood pressure (BP) changes

All 19 trials investigated the change in BP of patients after undergoing RSD at various time points throughout follow-up. BP measurements were either “office BP,” “Ambulatory or 24-h BP” or “mean systolic and diastolic BP”. Two studies reported no significant change in BP 3 months post-RSD [24, 27] but Ukena et al. [42] report a significant decrease in BP at 3 months. Nine studies recorded BP in patients 6 months post-RSD, of which five studies [26, 31, 36, 41, 42] reported a significant decrease in blood pressure; however, four studies reported no significant change from baseline [25, 27, 37, 40]. Two studies report BP outcomes at 9 months, both of which were reported to have seen a significant decrease of BP at that time point [28, 29]. At 12 months post-RSD, five studies reported a significantly lowered blood pressure [26, 28, 30–32]; however, two studies reported no significant change in blood pressure at that time point [24, 27].

As well as the standard follow-up schedule outlined above followed by a large plurality of studies, Tsioufis et al. [36] investigate a more short-term outcome and have found that RSD reduces BP significantly 1 month post-RSD despite Ukena et al. [39] reporting no significant change in BP after 1 month. Qiu et al. [34] report no change in BP at 7 days post-RSD. Evranos et al. [38] investigate BP beyond 12 months and report no change in BP at 15 months (Table 3).

### Renal outcomes

A total of seven included studies reported renal outcomes pre- and post-RSD [24–28, 31, 37, 38]. Seven studies recorded the estimated glomerular filtration rate (eGFR) [24–28, 31, 38]. Three studies [24, 25, 31] showed a significant increase (*p* < 0.05 or *p* < 0.001) in eGFR. All three of these studies reported significant increase in eGFR at 6 months post-RSD [24, 25, 31] and two studies reported a significant increase (*p* < 0.05 or *p* < 0.001) in eGFR at 12 months [25, 26]. Four studies reported a numerical increase or unchanged eGFR upon follow-up [27, 28, 31, 38]. The albumin to creatinine ratio (ACR) was reported in four studies [24, 25, 27, 31], of which three studies reported a significant decrease (*p* < 0.05 or *p* < 0.001) in the ACR [24, 25, 31] and a single study, Kiuchi et al. [27] reported a numerical decrease in the ACR at follow-up. Creatinine levels were reported in five studies [24–27, 37]. Three studies [24, 25, 31] reported a significant decrease (*p* < 0.05 or *p* < 0.001) in creatinine and a further two reported numerical decreases at 6 months [37] and 12 months [27] (Table 4).

**Table 4:** Outcomes pertaining to renal function and echocardiographic assessment

Author	Year	Renal outcomes	Echocardiographic findings
Steinberg [30]	2020	NR	In the RDN group, at 12 months, there was a significant decrease in atrial diameter (mm), resulting in a between group difference of − 0.5 (95% CI − 1.1 to − 0.1) (*p* < 0.01). The addition of renal denervation resulted in a significant decrease of intraventricular septal thickness (*p* < 0.001). There was no significant change in LVEF in both groups
Ukena [33]	2019	NR	NR
Feyz [31]	2018	Renal function remained unchanged at both 6- and 12-month follow-up, eGFR (ml/min) pre-RDN was 83 ± 20 vs. 86 ± 21 at 6 months (*p* = 0.23) and 86 ± 23 at 12 months (*p* = 0.14)	There were no significant increases or decreases in volumes and dimensions at 6 and 12 months, respectively
Kiuchi [26]	2018	There was a significant decrease in Cr. from 1.11 ± 0.12 at baseline to 1.03 ± 0.12 (*p* < 0.05) at 6 months and 0.97 ± 0.09 (*p* < 0.0001) at 12 months. eGFR showed a significant increase from 69.2 ± 6.7 to 76.2 ± 7.2 (*p* < 0.05) at 6 months and 81.8 ± 6.8 (*p* < 0.0001) at 12 months. The ACR showed a significant decrease from 75.0 ± 23.4 to 62.1 ± 21.3 (*p* < 0.05) at 6 months and 39.5 ± 15.5 (*p* < 0.0001)	NR
Jiang [37]	2018	Pre-RDN creatinine (µmol/L) 78.5 ± 23.2 decreased numerically to 69.5 ± 13.1 (*p* = 0.161)	NR
Romanov [32]	2016 (Made available in 2017)	NR	NR
Kiuchi [25]	2016	There was a significant decrease in Cr. from 1.3 ± 0.2 at baseline to 1.1 ± 0.2 (*p* = < 0.05) at 3 months, 1.1 ± 0.2 (*p* < 0.001) at 6 months and 12 months. For eGFR, there was a significant increase from 59.3 ± 13.3 at baseline to 62.5 ± 12.2 (*p* < 0.05) at 3 months, 64.9 ± 13.4 (*p* < 0.001) at 6 months and 65.7 ± 14 (*p* < 0.001) at 12 months. There was a significant decrease in the ACR from 85 (66–116.0) at baseline to 44.0 (31–74) (*p* < 0.05) at 3 months, 31.0 (21–53) (*p* < 0.001) at 6 months and 19.0 (11.5–32.5) (*p* < 0.001) at 12 months	In the PVI and RSD groups (*n* = 21), there was a significant increase in LVEF from 62.7 ± 6.6 at baseline to 65.8 ± 7.0 at 12 months (*p* = 0.0016). There was a significant decrease in LAD from 45.1 ± 3.2 at baseline to 42.9 ± 3.4 at 12 months (*p* = 0.0018). There was a significant decrease in LVIDd (mm) from 54.0 ± 3.0 at baseline to 51.6 ± 2.6 (*p* = 0.0001). There was a significant decrease in LVMI from 107.0 ± 13.5 at baseline to 97.9 ± 12.3 at 12 months (*p* = 0.0097)
Kiuchi [24]	2016	There was a significant decrease in Cr. from 1.53 ± 0.15 at baseline to 1.31 ± 0.11 (*p* < 0.0001) at 6 months. There was a significant increase in eGFR from 47.9 ± 6.8 at baseline to 59.0 ± 5.0 at 6 months (*p* < 0.0001). There was a significant decrease in the ACR from 90.0 ± 16 at baseline to 58.9 ± 20 (*p* < 0.0001)	In the PVI and RSD groups (*n* = 39) for the indexed left atrial volume (ml/m^2^), there was a significant decrease from 39.8 ± 9.4 to 36.1 ± 5.0 (*p* = 0.0331). For intraventricular septum thickness (mm), there was a numerical decrease from 9.0 ± 2.3 to 8.9 ± 1.6 (*p* = 0.8242). For LVPWT, there was a numerical decrease from 9.5 ± 1.8 to 9.3 ± 1.4 (*p* = 0.5855). For LVEF, there was a significant increase from 65.8 ± 12.8 to 70.5 ± 7.2 (*p* = 0.0492). For LVEDD, there was a significant decrease from 47 ± 4.3 to 44.2 ± 4.9 (*p* = 0.0090). For LVESD, there was a numerical decrease from 31.0 ± 6.9 to 29.1 ± 3.6 (*p* = 0.1315). For LVMI, there was a significant decrease from 94.3 ± 19.4 to 82.1 ± 17.5 (*p* = 0.0047)
Qiu [34]	2016	NR	NR
Evranos [38]	2016	The eGFR remained unchanged at baseline and follow-up baseline and 15-month follow-up	NR
Ukena [39]	2016	NR	NR
Kiuchi [27]	2016	There was a numerical decrease in Cr. from 0.8 ± 0.2 at baseline to 0.8 ± 0.1 (*p* = 0.1626) at 12 months. There was also a numerical increase in eGFR from 96.7 ± 17.7 at baseline to 98.5 ± 16.8 (*p* = 0.2797) at 12 months. There was a numerical decrease in the ACR from 20.4 (14.8–28.6) at baseline to 19.6 (13.2–23.2) at 12 months (*p* = 0.2035)	In the RSD group, for LVEDVI, there was a significant decrease from 87.7 ± 5.2 at baseline to 83.3 ± 5.5 at 12 months (*p* < 0.0001). There was a significant decrease in the LVESI from 32.8 ± 2.7 to 29.7 ± 2.5 (*p* < 0.0001). There was a significant decrease in LVMI from 81.5 ± 8.2 at baseline to 76.1 ± 7.4 at 12 months (*p* < 0.0001). There was a significant increase in LVEF from 68.1 ± 5.6 at baseline to 71.1 ± 5.2 (*p* = 0.0010) at 12 months
Schirmer [41]	2015	NR	LVMI showed a significant decrease from 61.5 ± 2.0 at baseline to 53.4 ± 1.5 (*p* < 0.001). LAVI showed a significant decrease from 34.4 ± 1.1 at baseline to 30.3 ± 0.9 at 6 months (*p* < 0.001). *E*_max_ (cm/s) showed a significant increase from 66.9 ± 2.6 at baseline to 72.9 ± 2.1 at 6 months (*p* = 0.013). Deceleration time (ms) showed a significant decrease from 252 ± 9 at baseline to 227 ± 6 at 6 months (*p* = 0.010). E' showed a significant increase from 6.6 ± 0.27 at baseline to 7.35 ± 0.28 (*p* = 0.011). E/A showed a significant increase from 0.89 ± 0.05 at baseline to 1.01 ± 0.06 at 6 months (*p* = 0.002)
Armaganijan [40]	2015	NR	NR
McLellan [35]	2015	NR	There was a numerical decrease in LVEDD from 48 ± 5 at baseline to 47 ± 4 at 6 months (*p* = 0.50). There was a numerical increase in LVESD from 29 ± 6 at baseline to 32 ± 6 at 6 months (*p* = 0.12). LVEF remained unchanged. LV mass showed a significant decrease from 215 ± 60 at baseline to 192 ± 50 (*p* = 0.05). The LVMI showed a numerical decrease from 106 ± 27 at baseline to 95 ± 24 (*p* = 0.06). There was a numerical decrease in intraventricular septum thickness (mm) from 13 ± 2 at baseline to 12 ± 2 at 6 months (*p* = 0.36). There was a significant decrease in posterior wall width from 11 ± 2 at baseline to 10 ± 2 at 6 months (*p* = 0.05). LA area showed a numerical increase from 22 ± 4 at baseline to 23 ± 5 at 6 months (*p* = 0.28). RA area showed a significant decrease from 18 ± 5 at baseline to 15 ± 5 (*p* < 0.01). *E*/*e*′ remained unchanged (*p* = 0.68). Deceleration time (ms) showed a numerical decrease from 253 ± 31 at baseline to 249 ± 61 (*p* = 0.84)
Tsioufis [36]	2014	NR	NR
Pokushalov [29]	2014	NR	NR
Ukena [42]	2013	NR	NR
Pokushalov [28]	2012	The eGFR at baseline and at 6-month follow-up remained unchanged at 78.0 ± 6.1 and 81 ± 4.6 (*p* = 0.42), respectively	Mean LV mass was reduced in the PVI and RDN groups (*n* = 13) by approximately 10%. The reduction in LV mass was due to the reduction of intraventricular septal, posterior wall and relative wall thickness. All results were statistically significant

*AF* atrial fibrillation, *ATP* anti-tachycardia pacing, *CKD* chronic kidney disease, *DBP* diastolic blood pressure, *HTN* hypertension, *NR* not reported, *PAC* premature atrial complex, *PVC* premature ventricular complex, *PVI* pulmonary vein isolation, *SBP* systolic blood pressure, *RCT* randomised clinical trial, *RSD* renal sympathetic denervation, *VF* ventricular fibrillation

Assessments of renal function: *ACR* albumin:creatinine ratio (mg/g), *Cr*. creatinine (mg/dL), *eGFR* estimated glomerular filtration rate (ml/min/1.73 m^2^). Echocardiographic parameters: *LVEF* left ventricular ejection fraction (Simpson %), *LVEDD* left ventricular end diastolic diameter (mm), *LVESD* left ventricular end systolic diameter (mm), *LVMI* left ventricular mass index (g/m^2^), *LAVI* left atrial volume index (ml/m^2^), *LAD* left atrial diameter (mm), *AD* right atrial diameter (mm), *LVIDd* end diastolic left ventricular internal dimension, *LVPWT* left ventricular wall posterior wall thickness (mm), *LVESI* left ventricular end systolic volume index (mm), *E*_*max*_ deceleration time (ms), *LV* left ventricular, *LA* left atrial, LA area (cm^2^),* E'* early diastolic mitral annular tissue velocity

### Complications, hospitalisation and quality of life

Fourteen included trials investigated the complications (general and procedure related) post-procedure. Ten studies report no complication following RSD. Three studies specifically stated the absence of “procedure related complications” [26, 28, 29] and the remaining seven studies state the absence of “complications” [24, 25, 27, 34, 37–39]. However, Steinberg et al. [30] reported seven procedural complications and Feyz et al. [31] reported one procedural complication. Although Armaganijan et al. [40] reports “no major complications”, one patient developed severe bradycardia during the procedure. Ukena et al. [42] reported 10 complications in their RSD treatment group, that included eight transient vagal reaction during RSD and two pseudoaneurysms of the femoral artery.

Only five studies collected data regarding hospitalisation post-RSD (Table 5). Four studies report that all their patients were hospitalised 24 h post-RSD [24–27]. Steinberg et al. [30] reported that a total of eight patients (5.2%) were hospitalised post-procedure for atrial fibrillation-related symptoms (*n* = 5), cardiac or vascular surgery (*n* = 2) and due to myocardial infarction (*n* = 1). Only Feyz et al. [5] report quality of life (QoL) to be significantly improved at 6 and 12 months following RSD.

**Table 5:** Post-procedure outcomes

Author	Year	Complication	Hospitalisation	Death
Steinberg [30]	2020	7—procedural complications	8 patients (5.2%) (*n* = 5 for AF-related symptoms, *n* = 2 for cardiac or vascular surgery, *n* = 1 for myocardial infarction) (absolute risk reduction, 7.0%; 95% CI 1.6–12.5%; *p* = 0.03) (less than *n* = 18 from PVI only group)	2(1.3%)—none related to ablation (*n* = 1 fatal myocardial infarction, *n* = 1 non-cardiac cause)
Ukena [33]	2019	NR	NR	NR
Feyz [31]	2018	1—peri-procedural complication was reported involving a renal artery dissection that resolved after balloon dilatation	NR	0
Kiuchi [26]	2018	0—no procedural complications	All patients were hospitalised on the ward for 24 h	NR
Jiang [37]	2018	0	NR	0
Romanov [32]	2016 (made available 2017)	NR	NR	NR
Kiuchi [25]	2016	0	The patients remained hospitalised in the ward for 24 h after the procedure	0
Kiuchi [24]	2016	0	After the procedure all patients remained hospitalised for a period of 24 h	NR
Qiu [34]	2016	0	NR	NR
Evranos [38]	2016	0	NR	1—decompensated heart failure
Ukena [39]	2016	0	NR	3—progressive heart failure, septic shock
Kiuchi [27]	2016	0	The patients remained hospitalised in the ward for 24 h after the procedure	NR
Schirmer [41]	2015	NR	NR	NR
Armaganijan [40]	2015	1—no major procedure-related complication but *n* = 1 patient developed severe bradycardia during RSD	NR	3—none were attributed to VA, *n* = 1 heart failure, *n* = 1 decompensated heart failure, *n* = 1 endocarditis
McLellan [35]	2015	NR	NR	NR
Tsioufis [36]	2014	NR	NR	NR
Pokushalov [29]	2014	0—no procedural complications	NR	NR
Ukena [42]	2013	10—*n* = 8 patients experienced a transient vagal reaction during RSD and *n* = 2 developed a pseudoaneurysm of the femoral artery	NR	NR
Pokushalov [28]	2012	0—no procedure-related complications	NR	NR

*AF* atrial fibrillation, *ATP* anti-tachycardia pacing, *CKD* chronic kidney disease, *DBP* diastolic blood pressure, *HTN* hypertension, *NR* not reported, *PAC* premature atrial complex, *PVC* premature ventricular complex, *PVI* pulmonary vein isolation, *SBP* systolic blood pressure, *RCT* randomised clinical trial, *RSD* renal sympathetic denervation, *VF* ventricular fibrillation

### Mortality

Of the seven studies that report death post-RSD, three studies report no death at all [25, 31, 37]. However, of the three, Feyz et al. [31] only report “no cases of cardiovascular death”. Steinberg et al. [30] and Evranos et al. [38] report two (none related to ablation) and one (decompensated heart failure) death(s) in their RSD groups, respectively. Ukena et al. [39] reports three deaths within 1 year of RSD due to progressive heart failure (*n* = 2) at 6 and 7 months post-RSD, and septic shock with respiratory failure (*n* = 1) 2 months post-RSD. Lastly, Armaganijan et al. [40] report three cases of death but none was attributable to ventricular arrhythmias (Table 5).

## Discussion

The role of RSD in attenuating cardiac arrhythmias lies in the link between the renal afferent and efferent sympathetic nervous system (SNS) fibres that modulate the central nervous system control of cardiac electrophysiological properties. The SNS increases HR and increases atrioventricular conduction which is potentially arrhythmogenic. RSD operates by dampening pathological SNS overstimulation. Moreover, the SNS innervates the kidneys, playing a major role in the pathophysiology of resistant hypertension via the renin–angiotensin–aldosterone system (RAAS) and sodium absorption. This supports the findings of recent studies that suggest this autonomic overdrive plays an important aetiological role in many cardiac arrhythmias such as AF and potentially an explanation of why it often coexists with hypertension. Recent evidence suggests that an overdrive of the SNS contributes to the “perception” of paroxysmal symptomatic AF episodes and hence it is promulgated that the dampening of SNS activity not only decreases BP but also may decrease the occurrence of such “perceived” arrhythmias particularly paroxysmal AF [43, 44]. Given that the autonomic nervous system innervates both atria and ventricles, it is postulated that RSD might have a role in treating arrhythmias of both atrial (i.e. AF) and ventricular origin (i.e. VT or VF) [45].

Hypertension has been shown to be the most relevant risk factor in the development of AF, which is itself the most common type of arrhythmia in the United Kingdom and set to grow in prevalence as the population ages [46, 47]. The link between the SNS activity, cardiac arrhythmias and hypertension is well documented and has been a target of interest for much research for numerous pharmacological and non-pharmacological interventions [48, 49]. Furthermore, first-line pharmacological treatments for hypertension such as angiotensin-converting enzyme (ACEi) inhibitors and angiotensin receptor blockers (ARBs) are also noted for their use in patients suffering from AF [48]. However, poor drug adherence [50] as well has the increased prevalence of treatment-resistant hypertension, a single intervention with long-term efficacy is seen as an increasingly attractive option for healthcare providers, researchers and patients. RSD has a controversial history regarding its use to lower BP of patients due to the findings of the SYMPLICITY HTN-3 trial [16]. However, redesigned so called ‘second generation’ studies such as SPYRAL HTN -ON, SPYRAL HTN-ON and RADIANCE SOLO have reported that RSD has been shown to reduce Office and Ambulatory BP in patients for up to 12 months [51]. However, though RSD has been shown to be effective in the short term (up to 12 months), there is still a paucity of data on the long-term outcomes of hypertensive patients. The largest study on the longer term outcome is the is the Global Simplicity Registry (GSR) a multi-centre study, which has reported data on more than 2500 patients up to 3 years post-procedure [52].

These extensive studies have, however, shown that RSD is a safe procedure for patients with no significant complications with sham or control groups. The majority of the studies in this systematic review had no complications. There was no mortality directly attributable to the procedure itself in any of the studies. Of note is the large variation in reporting of complications between the studies—the majority of complications were reported in two studies Steinberg et al. [30] and Ukena et al. [42]. The majority of complications were, however, benign with vasovagal effects being predominant. Only one arrhythmic complication (severe bradycardia) was seen, underlining the link between the SNS and cardiac electrophysiology.

Another safety concern surrounding RSD is the potentially deleterious effects on renal function. However, the safety of renal denervation has been shown in multiple studies such as Hering et al. [53] and Prasad et al. [54] where both found no significant alterations in renal function in patients with stage 3–4 kidney disease after renal denervation up to 12 and 24 months, respectively. Furthermore, with RSD, there was no significant difference in mortality or adverse outcomes in the studies that could be attributed to ventricular arrhythmias or the renal denervation procedure itself.

Quality of life scores, though initially may seem disappointing, likely do not reflect an accurate representation of the efficacy RSD on quality of life for cardiac rhythm disturbances—many of the trials were primarily driven for HTN and not for cardiac rhythm disturbances. The pre-existent rhythm disturbances may not be significantly impacting upon the baseline QoL making improvements hard to detect. HTN itself is largely asymptomatic, and therefore, QoL improvements are likely to be driven by reduced medication needs and contact with medical services in the studies where this was the primary end point.

The RSD procedure itself was technically more comparable. Similar radiofrequency energy use across the studies helps in comparison. Anticoagulation peri-procedurally was also largely with unfractionated heparin. However, ablation time was more variable (only one study used ablation < 30 s, all others > 60 s, though a significant number > 120 s). Thus, from a technical aspect, the largest point of difference is the ablation time (and therefore, total energy delivered) as well as the equipment used to deliver the energy. Ablation time may simply reflect operator familiarity with the anatomy to faithfully deliver the ablation to the target.

The effect of RSD on arrhythmic outcomes seems to be more pronounced. The atrial arrhythmia of primary concern in the studies was AF, with a secondary focus on atrial flutter. Flutter outcomes were not commented on between the two arms apart form in one study where there was a significant decrease in recurrence of atrial flutter. This is not unexpected as of the eight studies that measured outcomes RSD with concurrent ablation for AF/flutter, and all the protocols included empirical cavo-tricuspid isthmus line ablation if the patient had a history of atrial flutter, a procedure with a high success rate without adjunctive therapy. Therefore, it would not be expected to see significant recurrence, and therefore, reduction in recurrence of flutter regardless if RSD had been performed or not.

The outcomes for AF are worth considering carefully. Six studies measured pulmonary vein isolation (PVI) and compared it to PVI and RSD for the treatment of AF. The majority of these studies were in patients with a history of paroxysmal AF, a patient population in which PVI has greater success. However, two studies included patients with episodes of persistent AF as well as paroxysmal AF. Permanent AF was excluded, as by definition, these patients are not to be restored to sinus rhythm. In all the studies, there was a significant improvement in freedom from AF with adjunctive RSD treatment with PVI. Furthermore, the detection of AF was usually done with Holter monitoring, though at least half the studies used implantable devices to continuously monitor cardiac rhythms, which will improve detection rates of arrhythmias dramatically. The remaining four studies used Holter monitoring varying in duration from 24 h at prespecified times up to the 12-month follow-up period to 7 day Holter monitoring. In all these studies, there was statistically significant improvement in freedom from AF. The improvement in outcomes was consistent despite large variation in the ability to detect arrhythmias (i.e. the total time where surveillance for AF was conducted was variable between studies) suggesting a real effect rather than one driven by poor detection.

A plurality of studies documented similar improvements in PACs (premature atrial complexes) though this was not universal. Improvements in the ventricular response rates of patients with persistent AF were also noted. The results for ventricular arrhythmias in patients who underwent RSD were similarly poignant, with six of nine studies showing a reduction in ventricular arrhythmias of all kinds following RSD. In the three studies that showed no such effect, the total surveillance time was very low—Ukena [33] had a single 24-h Holter detection period at one follow-up point (6 months post-procedure) whilst McLellan [35] had a 7-day surveillance period at 6 months post-procedure. It is entirely feasible that this is not a long enough surveillance time to detect statistically significant changes in ventricular arrhythmias, especially considering the relative lower frequency of ventricular compared to atrial arrhythmias. The 6-month follow-up point to measure outcomes may initially seem shorter than the 12 months in other studies, though should be long enough to detect changes induced by ventricular remodelling according to the body of the literature in other pathologies where ventricular remodelling is observed such as heart failure. Thus, there seems to be a somewhat conflicting evidential basis for reductions in ventricular arrhythmic burden with RSD. Future studies will have to more carefully define the patient population enrolled into the studies and perhaps concentrate on patient with known IHD and higher burden of ventricular arrhythmias from the outset—this will allow detection of a change in ventricular arrhythmic burden in the patient population that is most at risk and allow studies to detect smaller changes with greater power.

The effect on heart rate is more mixed, with no clear consensus emerging. This may be due to large confounds such as with antiarrhythmic and anti-hypertension medication that may alter heart rate, a whole range of other physiological correlates including co-morbidities as well as the quality of the studies themselves.

There seems to be indications for the efficacy for RSD for blood pressure control with the majority of studies showing statistically lower blood pressure from 6 months onwards in both an ambulatory and office setting. BP control at earlier time points (1–3 months post-RSD) and long-term outcomes of BP control with RSD have not been extensively investigated with only one study investigating outcomes beyond 12 months. The effect of this improved control on pharmacotherapy cannot be determined due to the large variability in reporting in the studies, with in depth dosage and treatment regimens before and after RSD not being available to perform a comparison.

End-organ effects of RSD were explored in terms of both renal and cardiac outcomes. Renal denervation therapy has been used as an invasive but safe procedure to treat heart failure [55]. Importantly, a number of studies reported that RDN improves left ventricular ejection fraction (LVEF), an important prognostic marker and determinant of clinical management. Further echocardiographic measures such as septal wall thickness and ventricular mass indices were also reduced in a number of studies. However, there is conflicting evidence with some studies reporting no such effect. More trials need to be conducted to follow-up on these findings. Similarly, the lack of large and reliable studies was noted as an issue in Fukuta et al. [56], which noted that RSD was associated with a statistically significant increase in LV function (including LVEF) as well as a broader improvement [56]. Similarly, there is conflicting evidence for improvement in eGFR, though reassuringly there is no evidence for RSD being associated with deterioration in renal function even in patients with chronic kidney disease.

Pulmonary vein isolation (PVI) is used widely to treat symptomatic AF; however, its success rate is not optimal and there is a marked recurrence rate of arrhythmia is certain subgroup of patients. However, with the addition of RSD to PVI, the results of several RCTs and meta-analysis seem promising with regards to lowering the rate of AF recurrence significantly [11, 30, 57]. Studies have reported a role for RSD in other atrial and ventricular arrhythmia subtypes, and the majority of studies here demonstrate reduced ventricular arrhythmias. Importantly, however, these studies were not adequately controlled for concomitant antiarrhythmic drug use and a large number of patients were taking beta-blockers in the studies. Further studies are warranted to be able to draw firm conclusions.

In our analysis, it has proven difficult to pool results across studies with regards to the outcomes of RSD largely due to two reasons: (1) the small population size in studies. As RSD has yet to be performed as a standard treatment for arrhythmias, large, randomised studies have not been easy to conduct and the logistically feasible alternatives such as cohort studies in real-world settings have proven useful for investigating different outcomes for RSD. Arrhythmia outcomes in RSD studies often rely on subgroup analysis which are often underpowered with small sample sizes. (2) Large heterogeneity in procedural protocol, treatment regimen, care pathway and RSD equipment. Namely, the majority of studies used RSD in combination with PVI on the treatment arm, and therefore, highlighting the scarcity of evidence of the implications of using RSD in isolation for arrhythmias. Therefore, treatment outcomes across several institutions cannot be compared without acknowledging the fact that the differences mentioned have a role to play in patient outcomes.

This systematic review has several limitations that stem from the quality of studies included. (1) The review included a large number of unblinded, non-randomised observational studies. Data extracted from these cohort studies are potentially affected by biases due to the study design such as selection bias and allocation bias. (2) The generalisability and statistical power of the data is questionable given that the studies mostly do not have a large study population, some as few as 8. (3) The studies were also not powered sufficiently for us to perform subgroup analysis on the outcomes. (4) Most studies include patients with hypertension and form of arrhythmia, i.e. AF. Therefore, it can be argued that the hypothesis drawn based on hypertension patients cannot be extrapolated to patients without hypertension. (5) In some patients, antihypertensives and antiarrhythmic were used alongside RSD, and therefore, could have potentially influenced the true outcome of the procedure. (6) In some studies, the RSD procedure most notably total ablation power delivered, was not standardised and could have an impact on the efficacy of the treatment and outcomes. (7) The patient population in some trials are heterogenous and propensity scores were not matched to minimise confounding factors. (8) The inclusion of several types of arrhythmia as an outcome did not allow us to interpret the outcomes of each arrhythmia individually for a minuscule portion of the analysis. Lastly, the reporting of arrhythmia recurrence was variable which made it difficult for us to pool data and to quantify the difference from various studies for analysis.

To gain a more concrete and directional understanding on the true effects of RSD on cardiac arrhythmias, future studies should be conducted to investigate further the potential clinical efficacy, safety and benefits in arrhythmia patients in large-scale RCTs. These studies should be arrhythmia specific and have long-term outcome measures that go beyond a year post-RSD. The focus of these studies should initially be on AF patients with resistant hypertension as this is currently a major issue in clinical practice. Such studies may produce sufficient data, that might enable the safe, efficacious and minimally invasive RSD to be incorporated into clinical guidelines permitting its use in everyday clinical practice. Consensus on the most effective RSD protocols is also needed, therefore, enabling further research to be done on a multi-centre and international scale. With uniformity in the deliverance of RSD, more direct comparisons could be made, leading to a further therapeutic option in the clinician’s arsenal.

The role of RSD in attenuating AF is becoming more widely accepted when used with PVI [26, 28–32, 34]. Its potential in treating other arrhythmias has yet to be established but the evidence published till date report promising results.

## Data Availability

NA.

## Code availability (software application or custom code)

NA.
